# Fasciculation and Guidance of Spinal Motor Axons in the Absence of FGFR2 Signaling

**DOI:** 10.1371/journal.pone.0041095

**Published:** 2012-07-17

**Authors:** Rosa-Eva Huettl, Teresa Haehl, Andrea B. Huber

**Affiliations:** Institute of Developmental Genetics, Helmholtz Zentrum München – German Research Center for Environmental Health, Neuherberg, Germany; Virginia Commonwealth University Medical Center, United States of America

## Abstract

During development, fibroblast growth factors (FGF) are essential for early patterning events along the anterior-posterior axis, conferring positional identity to spinal motor neurons by activation of different Hox codes. In the periphery, signaling through one of four fibroblast growth factor receptors supports the development of the skeleton, as well as induction and maintenance of extremities. In previous studies, FGF receptor 2 (FGFR2) was found to interact with axon bound molecules involved in axon fasciculation and extension, thus rendering this receptor an interesting candidate for the promotion of proper peripheral innervation. However, while the involvement of FGFR2 in limb bud induction has been extensively studied, its role during axon elongation and formation of distinct nervous projections has not been addressed so far. We show here that motor neurons in the spinal cord express *FGFR2* and other family members during the establishment of motor connections to the forelimb and axial musculature. Employing a conditional genetic approach to selectively ablate *FGFR2* from motor neurons we found that the patterning of motor columns and the expression patterns of other FGF receptors and *Sema3A* in the motor columns of mutant embryos are not altered. In the absence of FGFR2 signaling, pathfinding of motor axons is intact, and also fasciculation, distal advancement of motor nerves and gross morphology and positioning of axonal projections are not altered. Our findings therefore show that FGFR2 is not required cell-autonomously in motor neurons during the formation of initial motor projections towards limb and axial musculature.

## Introduction

In the developing vertebrate organism, the establishment of functional neuronal networks presents a challenging endeavor: a large variety of functionally distinct neuronal subtypes needs to be generated and the formation of appropriate connections to their peripheral targets has to be precisely regulated. Neuronal localization and subsequent enactment of specific neuronal identities are defined already at early embryonic stages by dorso-ventral, medial-lateral, and rostro-caudal patterning mechanisms and consequential activation of transcription factors at defined positions along the neural tube [1]. The establishment of axonal projections from these neurons to distinct peripheral targets is then achieved in a stepwise process, comprising the correct exit of axons from the neural tube, adequate bundling with other axons and subsequent guidance by attractive or repulsive interactions of axon-bound receptors with their ligands in the environment [2].

Somatic motor neuron identity is assigned to neuronal precursors in the pMN domain through the stepwise activity of homeodomain transcription factors such as Pax6, Nkx6.1, Nkx6.2 and Olig2, whose expression is fine-tuned to the graded expression of morphogens like Sonic hedgehog (Shh) in the floorplate and notochord [3], [4]. In the lateral motor columns (LMC), where motor neurons that innervate limb musculature reside, downstream targets of these transcription factors confer the ability to motor axons to choose either dorsal or ventral pathways during limb innervation [5], [6]. Fibroblast growth factors (FGFs) play essential roles in the induction and anterior-posterior patterning of the neural plate, the local patterning of developing brain regions and in several steps of neurogenesis in ascidian and amphibian embryos, as well as in zebrafish and chicken (reviewed in [7]). While in mammals the role of FGFs in neural induction remains to be clearly demonstrated, they were shown to exert similar roles in rhombomere patterning and anterior-posterior patterning of the neural tube by regulation of intersegmental codes of homeobox transcription factor genes (Hox) in common vertebrate model organisms [8]–[11]. Graded expression of FGFs along the neural tube, for example, leads to activation of *Hox9*, whose expression coincides with thoracic motor neurons of the medial motor column (MMC), while at brachial levels, *Hox5*, *Hox6* and *Hox8* define the extent and the anterior and posterior borders of the LMC [12]–[14].

In mammals, the FGF family comprises 22 members, which interact with one of four highly related, partially functionally redundant thyrosine receptor kinases, the fibroblast growth factor receptors (FGFR; [15]). Alternative splicing of *FGFR1–3* generates receptor molecules containing different versions (a, b and c) of the immunoglobulin-like domain III, which is an essential determinant of ligand-binding specificity [16]. Among other tissues, these receptor molecules are expressed in various regions of the brain (*FGFR2* and *FGFR3*), the entire neural tube (*FGFR1*) or distinct regions in the spinal cord (*FGFR2* and *FGFR3*) of developing vertebrates [17]–[19]. *FGFR4* was shown to be expressed by zebrafish and amphibian neural tissues, however, its function in neural development remains unclear [20]–[22]. Ablation of either FGFR3 or FGFR4 affects the formation of the inner ear or lung development, but did not impair gastrulation or early patterning events of the embryo [23], [24]. Loss of either *FGFR1* or *FGFR2* results in early embryonal lethality, and ablation of receptor isoforms leads to severe morphological deficits, including growth retardation, malformation of extremities and defects in bone formation and ossification [25]. Furthermore, loss of FGFR1 signaling was shown to impair selective attraction of axial innervation towards thoracic target musculature [26], implying a role for FGF-FGFR signaling also in other axon guidance events. Ablation of *FGFR2* isoforms in particular results in malformations of the skeleton and deficits in limb bud initiation and maintenance [25], [27]–[29]. Accordingly, a number of human diseases such as Apert syndrome or Saethre-Chotzen-like syndrome, where craniofacial dysmorphologies are found along with defective morphology of the brain and limbs are associated with mutations in the *FGFR2* gene [25], [30]–[33]. Interestingly, next to these morphological deficits, murine embryos in which *FGFR2b* was ablated showed impairments in the development of the tooth epithelium and expression of the repulsive axon guidance cue Semaphorin 3A (Sema3A) was down-regulated, leading to deficits in dental axon patterning [34]. Previous studies in chicken embryos showed that intrinsic *Sema3A* expression in spinal motor neurons is essential for fine-tuning of axonal sensitivity to extrinsic sources of the guidance cue and contributes both to correct pathfinding and fasciculation of motor projections [35]. Which factors distinctly regulate *Sema3A* expression in these neuronal cells, however, still needs to be determined. Ablation of *FGFR2* in motor neurons was shown to lead to a transient deficit in presynaptic distribution of synaptic vesicles during late embryonal and early postnatal development [36], however, selective migration of motor axons to their target musculature was not analyzed up to now. During early embryonic development, FGFR2 is co-localized with neural cell adhesion molecule (NCAM), a key modulator of axonal growth and fasciculation in the developing brain which was observed to activate FGFR2 signaling and its downstream pathways [37]. *In vitro* studies where the function of FGFR2 was blocked showed a reduction of the growth promoting effect of N-cadherin (N-Cad, [38]), thus suggesting a role for the FGF receptor in axonal patterning, fasciculation and growth during innervation of the developing limbs.

In this study we sought to determine whether specific elimination of *FGFR2* from spinal motor neurons impacts on motor axon guidance decisions and axonal patterning to the periphery. We show that *FGFR2* is expressed by spinal motor neurons at developmental time points when axons are sent out to their peripheral targets. Using genetic tools to analyze the role of FGFR2 specifically in motor neurons for fasciculation and axonal growth promotion we find that signaling by this FGF receptor is not involved in the regulation of motor neuron-intrinsic *Sema3A* expression and the establishment of precisely bundled motor trajectories to the vertebrate forelimb. Furthermore, we demonstrate that FGFR2 signaling is dispensable in motor neurons for correct pathfinding of spinal motor axons to the distal limb. Our data therefore show that while FGFR2 is essential for the formation of vertebrate extremities, the reliable formation of axonal networks enabling locomotion depends on distinct mechanisms.

## Results

### *FGFR2* is Expressed by Spinal Motor Neurons of the Brachial LMC at Developmental Time Points of Forelimb Innervation

Among other tasks in neural induction and embryonic patterning, graded FGF-FGFR signaling along the spinal cord plays a role in the definition of the columnar identity of motor neurons within the ventral horn by activation of special Hox gene clusters [12]–[14]. Next to its important functions for bone formation and limb bud induction [25], [27], [28], FGFR2 was found to co-localize with NCAM, a key regulator of axon growth and fasciculation, which was shown to activate downstream signaling functions of the FGF receptor [37] already during very early embryonic development. Furthermore, *in vitro* studies suggest that crosstalk of axonal FGFR2 with N-Cad enhances axonal outgrowth [38]. Therefore, FGFR2 presents an interesting candidate for mediation of axon fasciculation and promotion of axonal growth.

**Figure 1:**
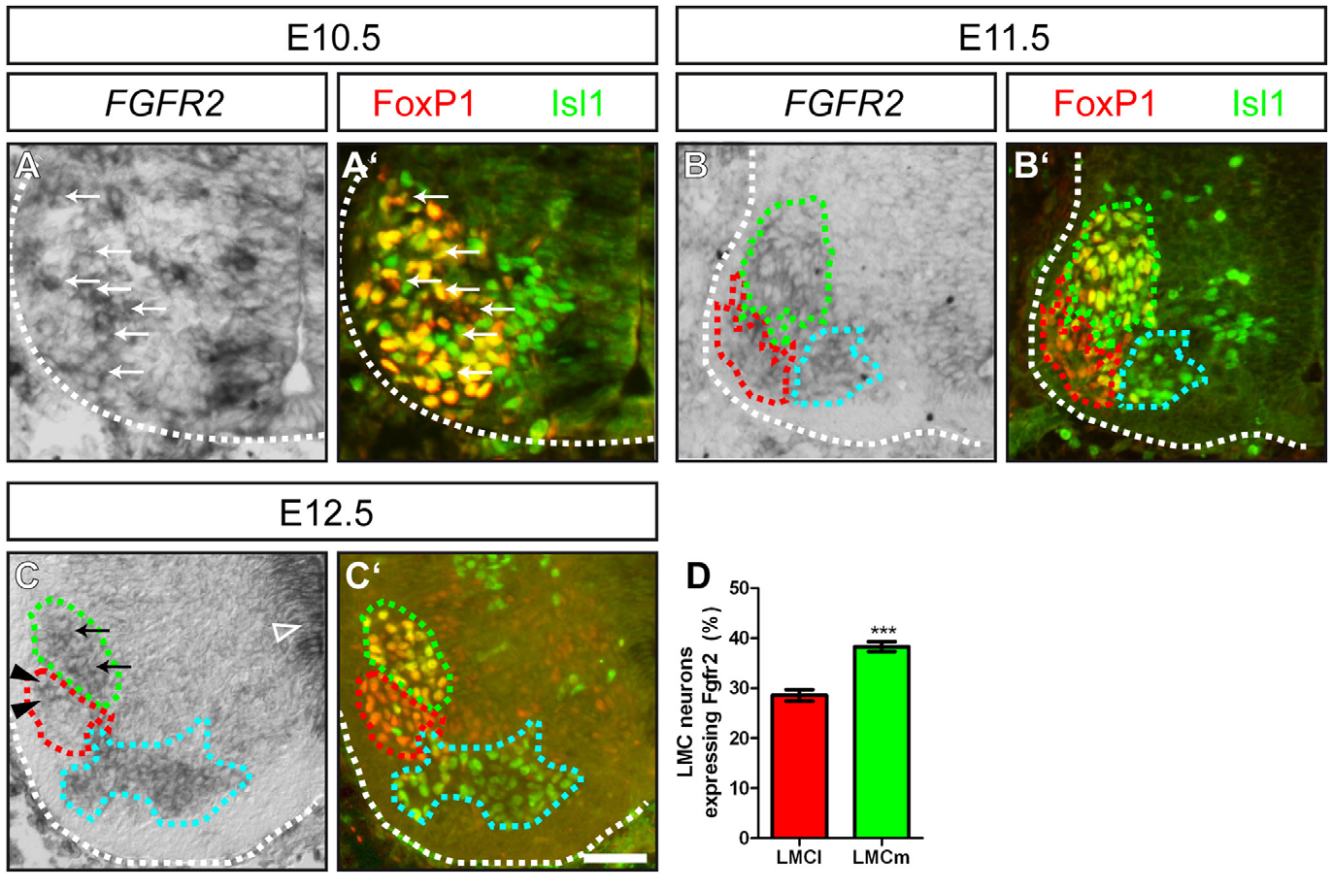
*FGFR2* is expressed in motor neurons of the LMC during forelimb innervation. (**A**, **A**’) At E10.5, spinal motor neurons in the ventral horn of the brachial spinal cord that will form the medial and lateral aspect of the LMC are identified by FoxP1 and/or Isl1 immunohistochemistry. A subset of these motor neurons shows expression of FGFR2 (arrows). (**B**, **B**’) At E11.5, motor neurons have segregated into two distinct sub-columns of the LMC; namely the LMCm (FoxP1^+^/Isl1^+^, green dashed line) and the LMCl (FoxP1^+^/Isl1^−^, red dashed line). *FGFR2* mRNA is found in the LMC and MMC (FoxP1^−/^Isl1^+^, cyan dashed line). (**C**, **C**’) *In situ* hybridization against *FGFR2* shows a higher number of motor neurons that express the FGF receptor in the LMCm (FoxP1^+^/Isl1^+^, green dashed line, arrows) when compared to dorsally projecting motor neurons of the LMCl (FoxP1^+^/Isl1^−^, red dashed line). (**D**) Quantification of *FGFR2* mRNA expression in motor neurons of the LMCm and LMCl showed a significantly higher number of ventrally projecting motor neurons that expressed the FGF receptor. Scale bar in (**C**’) equals 25 µm for (**A**), 40 µm for (**B**) and 50 µm for (**C**).

We investigated the expression pattern of the FGF receptor in the spinal cord of mouse embryos at the developmental time points when axons are extending to peripheral targets by *in situ* hybridization. Already at E10.5, when neurons of the LMC (characterized by FoxP1 expression) are not yet subdivided into the medial and lateral columns of the LMC, expression of *FGFR2* was observed in spinal motor neurons (arrows in Fig. 1A, A’). One day later, at E11.5, motor neurons that will innervate distinct ventral or dorsal target musculature in the developing extremities have clustered in the medial LMC (LMCm) and the lateral LMC (LMCl), respectively. For distinction of these columns we used antibody staining against Isl1, identifying LMCm motor neurons as FoxP1^+^/Isl1^+^ (green dashed line in Fig. 1B’), while LMCl motor neurons are characterized by FoxP1 expression in absence of Isl1 (red dashed line in Fig. 1B’). Expression of *FGFR2* was observed in both motor neuron populations (green and red dashed lines in Fig. 1B), as well as in neurons of the medial motor column (cyan dashed line in Fig. 1B, B’). At E12.5, we found a robust expression of *FGFR2* in the ventricular zone of the spinal cord (empty arrowhead in Fig. 1C), but also in the lateral and medial aspects of the LMC (arrowheads and arrows in Fig. 1C, respectively). When we quantified the number of *FGFR2* expressing cells in the two subdivisions of the LMC, we found that 38,29%+/−0,19 SEM of ventrally projecting LMCm neurons co-expressed the FGF receptor. In the lateral aspect of the LMC, 28,52%+/−0,52 SEM of dorsally projecting neurons were positive for *FGFR2* mRNA. (Fig. 1D, p≤0,001). Thus, 1,3 fold more ventrally projecting motor neurons express the FGF receptor when compared to LMCl neurons.

These findings show that *FGFR2* is expressed in a differential manner developing motor neurons as they extend their axons for targeted innervation of peripheral limb musculature.

### Conditional Ablation of *FGFR2* in Motor Neurons by *Olig2-Cre*

**Figure 2:**
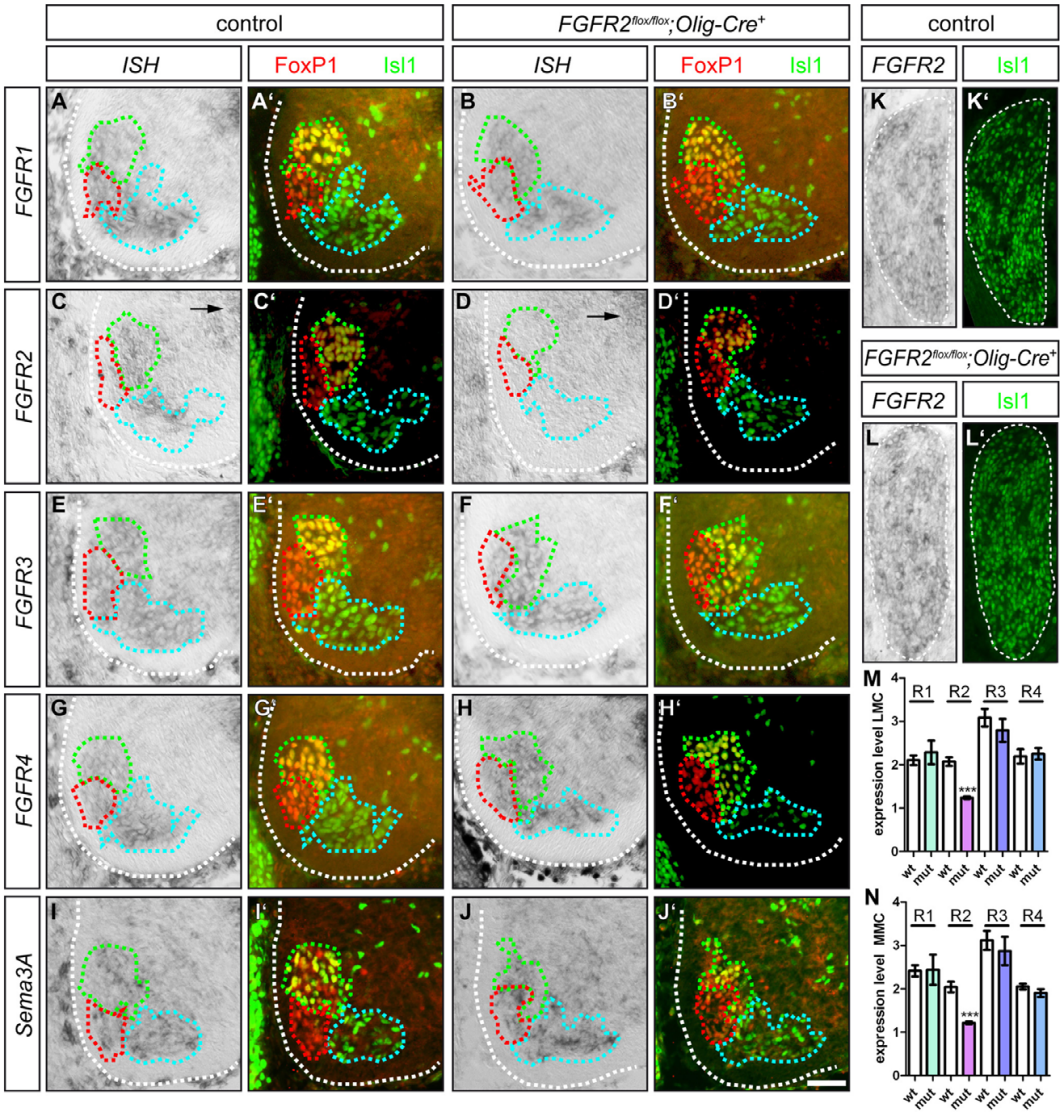
Expression analysis of *FGFR1–4* and *Sema3A*. (**A**’–**J**’) The subdivisions of the LMC and the MMC are identified by immunohistochemistry against FoxP1 and Isl1. Motor neurons in the LMCm are FoxP1^+^/Isl1^+^ (green dashed lines), LMCl motor neurons are FoxP1^+^/Isl1^−^ (red dashed lines) and MMC motor neurons are FoxP1^−^/Isl1^+^ (cyan dashed lines). (**A**, **B**) *FGFR1* is expressed by motor neurons in the LMCm, LMCl and MMC of control and *FGFR2^flox/flox^;Olig2-Cre^+^* mutant embryos, respectively. (**C**) *FGFR2* mRNA is found in motor neurons of the LMCm and subpopulations of LMCl and MMC motor neurons in control embryos. (**D**) Expression of Cre recombinase under the *Olig2* promotor tissue-specifically ablates *FGFR2* expression in motor neurons of the LMC and MMC, while in the ventricular zone *FGFR2* mRNA is still detected (compare arrows in **C** and **D**). (**E**, **F**) *FGFR3* mRNA is detected in motor neurons of the LMC and MMC in both control and *FGFR2^flox/flox^;Olig2-Cre^+^* mutant embryos. (**G**, **H**) *In situ* hybridization against *FGFR4* shows expression of the FGF receptor gene in the ventral horn of the spinal cord of control and *FGFR2^flox/flox^;Olig2-Cre^+^* mutant embryos. (**I**) *Sema3A* is expressed by motor neurons in the LMCm, LMCl and MMC, respectively, in control embryos. (**J**) *Sema3A* is expressed by motor neurons of the LMCm, LMCl and MMC, respectively, in *FGFR2^flox/flox^;Olig2-Cre^+^* mutant embryos. (**K**, **L**) Expression of *FGFR2* in sensory neurons is not affected by ablation of *FGFR2* by *Olig2-Cre*. (**K**’, **L**’) Immunohistochemistry against Isl-1/2 to illustrate sensory neurons in the DRG. (**M**) Quantification of expression levels reveals a significant decrease of *FGFR2 in situ* hybridization signal in the LMC of *FGFR2^flox/flox^;Olig2-Cre^+^* mutant embryos, while expression levels of *FGFR1, FGFR3* and *FGFR4* in the LMC remain unchanged (p*^FGFR1^* = 0,52; p*^FGFR3^* = 0,45, p*^FGFR4^* = 0,78). (**N**) Also in the MMC, expression levels of *FGFR1, FGFR3* and *FRGR4* remain unchanged upon loss of *FGFR2* in motor neurons, while a significant decrease of *FGFR2 in situ* hybridization signal is observed in *FGFR2^flox/flox^;Olig2-Cre^+^* mutant embryos when compared to control littermates (p*^FGFR1^* = 0,95; p*^FGFR3^* = 0,60, p*^FGFR4^* = 0,20). Scale bar in **J**’ equals 45 µm for all panels.

**Figure 3:**
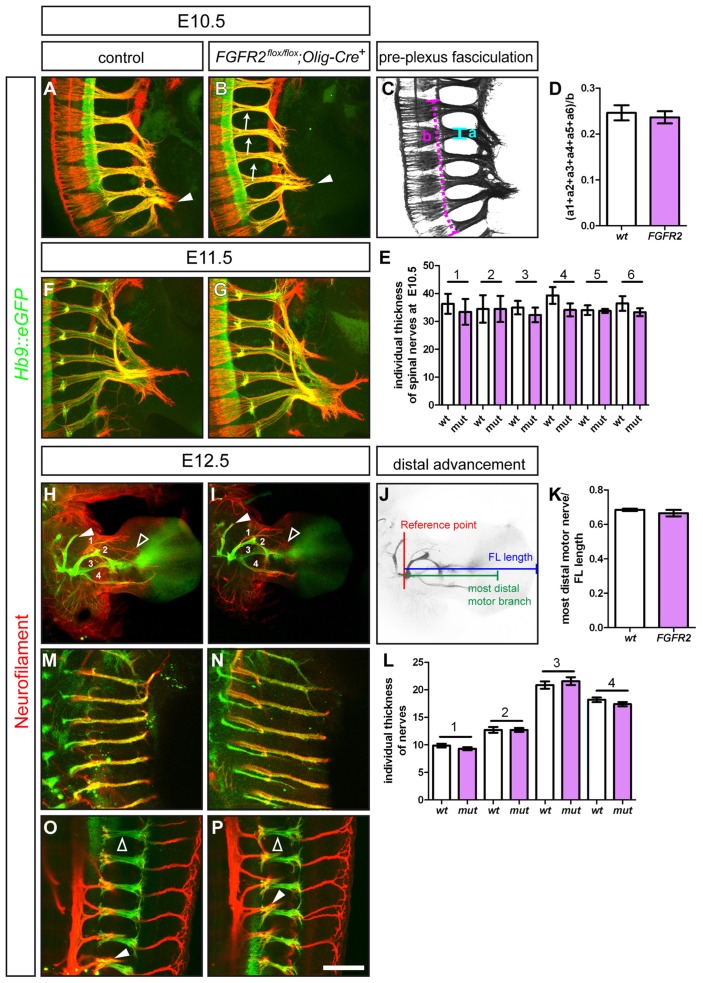
Ablation of *FGFR2* from motor neurons does not impair fasciculation, extension and gross morphology of nerve projections. Immunohistochemical staining of wholemount embryo preparations against *Hb9::eGFP* (green, motor nerves) and Neurofilament (red, motor and sensory nerves). At E10.5, tightly fasciculated spinal nerves have formed the brachial plexus at the base of the limb of control embryos (**A**) and in *FGFR2^flox/flox^;Olig2-Cre^+^* mutant embryos (**B**). (**C, D**) Quantification of the pre-plexus fasciculation of the 6 spinal nerves that form the brachial plexus shows no differences between control (0.25±0.017 SEM) and *FGFR2^flox/flox^;Olig2-Cre^+^* mutant embryos (0.24±0.013, p = 0,65). (**E**) Quantification of the individual thickness of spinal nerve branches that contribute fibers to the brachial plexus showed no significant difference between control and mutant embryos (p^1^ = 0,63, p^2^ = 0,99, p^3^ = 0,47, p^4^ = 0,20, p^5^ = 0,89, p^6^ = 0,32). (**F, G**) At E11.5, both in control and mutant embryos, first target specific fascicles have entered the limb mesenchyme. (**H**) Motor and sensory innervation of control embryo forelimbs. 1 =  branch of the radial nerve, 2 =  radial nerve, 3 =  median nerve, 4 =  ulnar nerve. (**I**) Gross morphology of motor and sensory innervation to the forelimb is not altered in embryos where *FGFR2* was ablated in motor neurons by *Olig2-Cre*. (**J, K**) The distal advancement of the median nerve is not impaired in *FGFR2^flox/flox^;Olig2-Cre^+^* mutant embryos (0.67±0.02) when compared to control littermates (0.68±0.01, p = 0,36). (**L**) Quantification of the individual thickness of the 4 major motor nerves shows not significant differences between control and mutant embryos in fasciculation (p^1^ = 0,24, p^2^ = 0,99, p^3^ = 0,47, p^4^ = 0,19). Innervation of intercostal muscles at thoracic levels forms tightly fasciculated nerve branches in control (**M**) and *FGFR2^flox/flox^;Olig2-Cre^+^* mutant embryos (**N**). Also innervation of epaxial musculature by the ascending branch (empty arrowheads) of MMC projections is established normally in *FGFR2^flox/flox^;Olig2-Cre^+^* mutant embryos (**P**) when compared to control embryos (**O,** arrowhead points to descending branch which innervates intercostal musculature). Scale bar in (**P**) equals 100 µm for **A, B, F** and **G,** 500 µm for **H** and **I,** and 200 µm for M and N, and 100 µm for **O** and **P.**

As a null mutation of *FGFR2* in the entire organism is lethal already at very early embryonal stages [39], we employed a conditional approach to selectively remove the receptor (*FGFR2^flox/flox^*, [40]) from motor neurons by tissue specific activation of Cre recombinase driven by the *Olig2* promotor [41]. *Olig2-Cre* is expressed by all somatic motor neurons starting as early as E8.5 and causes a deletion of exon 5 of *FGFR2,* which leads to a stop codon in the extracellular domain within exon 6. *In situ* hybridization against *FGFR2* shows expression in motor neurons of the brachial LMC and the MMC of control embryos, as well as in the ventricular zone of the spinal cord (Fig. 2B). If *FGFR2* was ablated by *Olig2-Cre*, motor neurons of the LMC and MMC (identified by FoxP1 and Isl1 immunohistochemistry) are devoid of *FGFR2* mRNA (Fig. 2D, D’). These findings were corroborated by analyses of the expression levels of *FGFR2* in motor neurons of the LMC and MMC, showing a significant decrease of the *in situ* hybridization signal in *FGFR2^flox/flox^;Olig2-Cre^+^* mutant embryos, when compared to control littermates (Fig. 3M, N, p^LMC^≤0,001, p^MMC^≤0,001). As expected, expression of *FGFR2* in the ventricular zone is not affected in *FGFR2^flox/flox^;Olig2-Cre^+^* mutant embryos (arrow in Fig. 2D). Also sensory neurons in the DRG still express the FGF receptor upon *Olig2-Cre*-mediated excision of exon 5 in spinal motor neurons (Fig. 2K, L).

As FGFs are known to interact with more than one FGF receptor, and FGFRs show a functional redundancy among each other [42], we also investigated the expression of the other three family members of the FGF receptors in the developing spinal cord. On sections of control embryos, expression of *FGFR1*, *FGFR3* and *FGFR4* was found in motor neurons of the LMCm (green dashed line, Fig. 2A, E, G), in motor neurons that project to dorsal limb musculature (red dashed line, Fig. 2A, E, G) and motor neurons of the MMC, which innervate axial musculature (cyan dashed line, Fig. 2A, E, G). In embryos where *FGFR2* was ablated in spinal motor neurons by *Olig2-Cre*, the expression pattern of the other three FGF receptors was not altered in motor neurons when compared to control littermates (Fig. 2B, F and H). These findings are corroborated by a detailed analysis of the expression levels of *FGFR1, FGFR3* and *FGFR4* in the LMC and MMC of control and *FGFR2^flox/flox^;Olig2-Cre^+^* mutant embryos: We found no significant changes in the expression levels of the three FGF receptors in the LMC and MMC of mutant embryos when compared to control littermates (Fig. 3M, N).

Ablation of *FGFR2b* in murine embryos was shown to down-regulate *Sema3A* expression in the tooth epithelium and cause patterning deficits of trigeminal dental axons [34]. Intrinsic *Sema3A* expression in spinal motor neurons fine-tunes axonal sensitivity to extrinsic *Sema3A* sources, thereby contributing to both correct pathfinding and fasciculation of motor projections [35]. Which factors regulate *Sema3A* expression in spinal motor neurons, however, is not known. We performed *in situ* hybridization against *Sema3A* on sections of *FGFR2^flox/flox^;Olig2-Cre^+^* mutant embryos to analyze whether loss of signaling via this FGF receptor specifically in motor neurons leads to a similar de-regulation of *Sema3A* expression as it was shown for embryos where *FGFR2b* was eliminated in the entire organism. In control embryos, motor neurons of the LMCm, LMCl and MMC showed expression of the repulsive axon guidance cue (Fig. 2I). In embryos where *FGFR2* was ablated by *Olig2-Cre*, motor neurons in the sub columns of the LMC as well as the MMC showed a comparable *Sema3A* expression (Fig. 2J).

These findings show that *Olig2-Cre* successfully removes *FGFR2* from somatic motor neurons of the LMC and MMC at brachial levels, while it does not target sensory neurons in the DRG. Expression of *Sema3A* in spinal motor neurons was not altered upon elimination of the FGF receptor from spinal motor neurons. Moreover, expression of the remaining FGF receptors in the spinal cord was not altered upon loss of *FGFR2* in spinal motor neurons. Furthermore, our data demonstrate that ablation of *FGFR2* in spinal motor neurons does not impair the positioning of dorsally and ventrally projecting motor neurons within the LMC.

### Conditional Ablation of *FGFR2* in Motor Neurons does not Alter Fasciculation and Growth Patterning of Motor Nerves

To assess whether ablation of *FGFR2* from motor neurons might cause any deficits in the fasciculation of motor axons that project to distal forelimb musculature, we analyzed wholemount embryo preparations: To distinguish sensory from motor axons, we crossed the *FGFR2^flox/flox^; Olig2-Cre* mouse lines to the *Hb9::eGFP* line, where expression of GFP is activated in all motor neurons [43], and performed fluorescent immunohistochemistry against Neurofilament (sensory axons, in the absence of GFP) and GFP (motor axons). At E10.5, motor and sensory axons, which project as tightly fasciculated spinal nerves before the plexus region, have reached the dorsal-ventral choice point at the base of the embryonic limb, but not yet navigated through it (Fig. 3A). As the *Olig2-Cre* line ablates the FGF receptor already before first axonal extensions are established [44], possible effects on axon growth and fasciculation thus might already be observed at early developmental stages. In *FGFR2^flox/flox^;Olig2-Cre^+^* mutant embryos we found that motor and sensory axons formed spinal nerves and correctly projected into the plexus region at the base of the limb and formed a normal brachial plexus (Fig. 3B). To determine whether fasciculation of the motor axons within the spinal nerves was affected by loss of *FGFR2* we calculated a fasciculation coefficient and found no significant differences in the fasciculation before the plexus region (Fig. 3C, D). Also the individual thickness of spinal nerves contributing to the brachial plexus did not vary between control and *FGFR2^flox/flox^;Olig2-Cre^+^* mutant embryos (Fig. 3E). At E11.5, in control embryos the first target specific axon bundles have formed and started to project towards the distal limb (Fig. 3F). In embryos where *FGFR2* was ablated in motor neurons, this formation of distinct sensory-motor projections to the specific targets in the limb still takes place and leads to the establishment of rami indistinguishable from the situation in control embryos (Fig. 3G). At E12.5, motor nerves have formed four individual nerve branches in the distal forelimb in wildtype embryos (Fig. 3H). Investigation of the growth patterning of these four major nerve branches of *FGFR2^flox/flox^;Olig2-Cre^+^* mutant embryos showed no obvious differences in the formation of these four nerve branches and the gross morphology of motor forelimb innervation, when compared to control embryos (Fig. 3H, I). Measurement of the individual thickness of the four major motor nerves contributing to forelimb innervation showed no increased diameter of the nerves and thus displayed no alterations in fasciculation of these nerve trunks (Fig. 3L). Sensory innervation of the forelimb is not affected by ablation of *FGFR2* in motor neurons when compared to littermate controls (empty arrowhead in Fig. 3H, I).

As FGFR2-N-Cad interaction was implicated to enhance axonal outgrowth, we analyzed whether loss of FGFR2 signaling in motor nerves impaired the extension of motor axons into the developing forelimb. We quantified the distal advancement of the median nerve (3) into the palm of the embryonic forelimb by correlating the length of the distalmost motor nerve branch to the length of the limb. We found no significant differences in the extension of this ventrally projecting nerve when comparing the distal advancement to littermate controls (Fig. 3J, K).

We showed that *Olig2-Cre* ablates *FGFR2* from all spinal motor neurons (Fig. 2D). Therefore, we also investigated the innervation of the intercostal and epaxial musculature from motor neurons in the MMC. In control embryos, motor axons form tightly fasciculated descending intercostal nerve branches at thoracic levels, with no crossings that might indicate pathfinding errors of MMC axons between the main fascicles (Fig. 3M). In embryos where *FGFR2* was eliminated from motor neurons we found similar results: motor axons from the MMC innervating intercostal musculature extend normally as tight fascicles between the ribs without aberrant connections between the distinct nerves (Fig. 3N). The ascending branches of MMC motor neurons innervate epaxial muscles of the back and form fasciculated nerve trunks at thoracic levels in control embryos (Fig. 3O). In embryos where *FGFR2* was ablated in somatic motor neurons by *Olig2-Cre*, we found no altered formation of epaxial motor branches to dorsal trunk musculature: MMC axons were fasciculated and formed branches as observed in control embryos, with no aberrant crossings or defasciculated fibers (Fig. 3P).

These findings argue for an only subordinate, if any, role of *FGFR2* in motor neurons for motor axon fasciculation and extension, and peripheral nerve patterning during embryonic development.

### Correct Pathfinding Decisions in Absence of *FGFR2* in Motor Neurons

**Figure 4:**
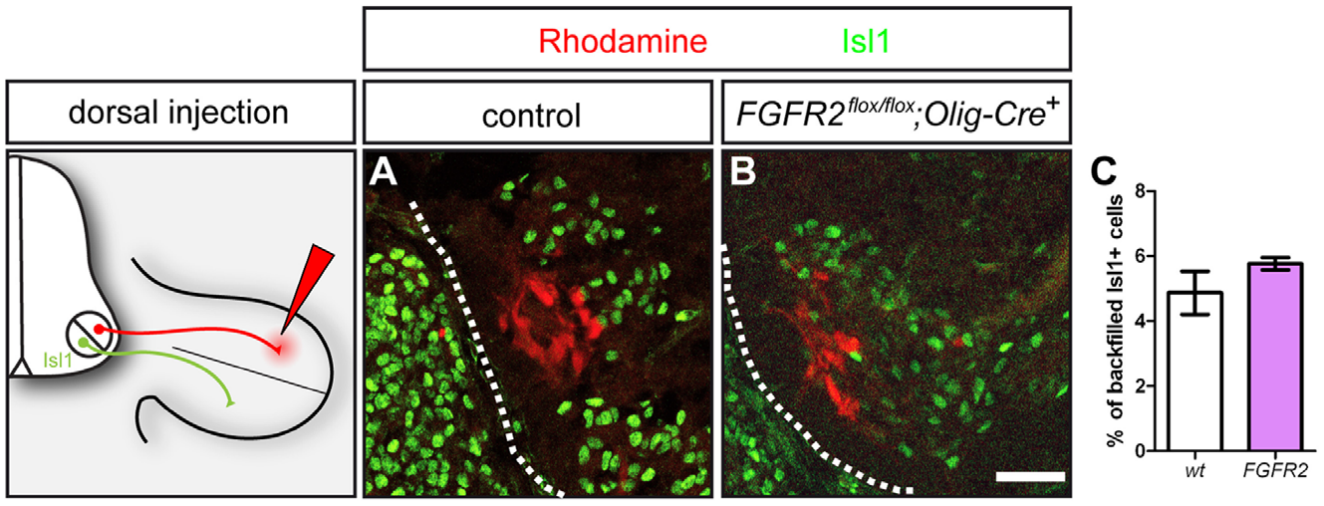
Guidance decision of ventrally projecting motor axons is not affected by loss of FGFR2 signaling in motor neurons. (**A**) Retrograde tracing with dextrane-conjugated Rhodamine from dorsal limb musculature labels Isl1^−^ motor neurons in the LMCl of control embryos. (**B**) Retrograde tracing from dorsal limb musculature labels Isl1^−^ motor neurons in the LMCl, while no Isl-1^+^ motor in the LMCm show a Rhodamine labeling. (**C**) Quantification of misprojecting, Isl1^+^/Rhodamine^+^ motor neurons after retrograde tracing from dorsal limb musculature shows no significant differences between control and *FGFR2^flox/flox^;Olig2-Cre^+^* mutant embryos. Scale bar in **B** equals 50 µm.

The establishment of precisely wired sensory and motor projections into the periphery requires correct polarized outgrowth of axons from differentiated neurons, subsequent axon pathfinding towards the target region, and the recognition of the appropriate synaptic partner. Over the past two decades, different adhesion molecules and guidance cues have been identified that are involved in mediating the dorsal-ventral guidance decision of motor axons [2]. Spatio-temporally controlled expression of guidance molecules in the environment and the activation of specific receptors on growth cone at the leading edge of the elongating axon leads to the activation of signal transduction pathways that activate cytoskeletal reorganizations governing axonal elongation, turning, or retraction (reviewed in [45]). Even though ablation of *FGFR2* in motor neurons does not obviously affect fasciculation and gross patterning of peripheral motor projections, axons might still be misguided at specific choice points. We found that *FGFR2* is differentially expressed within a higher number of ventrally projecting motor neurons of the LMCm expressing the FGF receptor. We therefore retrogradely labeled motor neurons projecting to dorsal limb musculature by injection of dextran-coupled Rhodamine into the dorsal limb of E12.5 embryos (Fig. 4A). In control embryos virtually all neurons correctly project to the ventral limb: we found that only 4,87%+/−0,66 SEM motor neurons that were backfilled from dorsal limb muscles were also Isl1-positive and thus represent misprojecting LMCm neurons. In *FGFR2^flox/flox^;Olig2-Cre^+^* mutant embryos, the incidence of misprojecting LMCm neurons was very similar, with only 5,76%+/−0,19 SEM of neurons of the LMCm that misrouted their axons to dorsal limb musculature (Fig. 4B, C, p = 0,26).

Therefore, conditional ablation of *FGFR2* in motor neurons does not lead to a significant increase of errors in the dorsal-ventral guidance decisions when compared to wildtype littermates.

## Discussion

Tightly regulated FGF-FGFR signaling along the rostro-caudal axis plays important roles in patterning of the developing embryo and activation of defined Hox genes that designate columnar identity of motor neurons in the spinal cord [12]–[14]. Isoforms of FGFR2 act upstream of Shh and govern limb bud induction, development and maintenance of the limb by interaction with FGF8 and FGF10 in the apical ectodermal ridge at the tip of the limb, but also critically contribute to growth and ossification of the bones [25], [27]–[29], [46]. The role of *FGFR2* for the formation and patterning of nervous projections into the limbs, however, has not been assessed up to now. Already during early embryonal development, FGFR2 was shown to be co-localized with NCAM, a modulator of axonal growth and fasciculation in the developing brain, which was observed to activate FGFR2 signaling and its downstream pathways [37]. At spinal levels, interaction of NCAM on motor axons with PSA secreted by motor axons to the surrounding tissue contributes to motor axon sorting and selective fasciculation of nerves before they grow into the distal limbs [47], [48]. In *in vitro* experiments, where FGFR2 signaling on motor axons was abolished, the growth promoting effect of N-Cad on axonal projections was blocked [38]. These findings correlate with experiments in early *Xenopus laevis* where expression of a dominant negative FGF receptor not only reduced the extension of retinal axons on N-Cad substrates [49], but also impaired target recognition of retinal ganglion cells [50]. Axonal pathfinding in the visual system and in the peripheral nervous system rely on similar guidance mechanisms [2], [51]. Therefore, these data in combination with findings of FGFR2 signaling regulating the expression of the repulsive axon guidance cue *Sema3A* during innervation of the teeth by trigeminal axons [34] may suggest a role for the FGF receptor in axon fasciculation, extension and guidance.

Using *in situ* hybridization we confirmed that *FGFR2* is expressed in somatic motor neurons during the period of axon extension and fasciculation when important choice points have to be navigated. At E12.5, we found that *FGFR2* is expressed differentially in the LMC, with higher levels in the medial aspect. Even though we did not find any dorsal-ventral guidance defects of LMCm axons projecting to the forelimb, we cannot exclude minor pathfinding errors of lateral LMC neurons, which are expressing considerably lower levels of FGFR2. In light of our careful and detailed analysis of fasciculation of peripheral nerves, timing of growth and positioning of specific rami in the forelimb that did not reveal any aberrations if *FGFR2* was ablated from spinal motor neurons, this appears unlikely.

During development, loss of either *FGFR1* or *FGFR2* leads to truncated limbs, or failure of limb bud induction, respectively, as survival and growth promoting signals from the same set of FGFs, namely FGF4, FGF8 and FGF10, in the progress zone and apical ectodermal ridge of the developing limb are no longer transferred [29]. As these FGFs present the most likely ligands for axon bound FGFR2 to facilitate axon guidance events, compensatory regulatory mechanisms by distinct FGF receptors might govern correct fasciculation and nerve growth. It was shown for the generation of oligodendrocyte precursors (OLPs) in the embryonic ventral forebrain, that ablation of either *FGFR1* or *FGFR2* resulted in a reduction of OLPs, however, only removal of both receptors resulted in complete absence of these precursor cells [42], showing a functional redundancy of the highly related FGF receptors. Accordingly, only combinatorial elimination of *FGFR1* and either *FGFR2* or *FGFR3* impaired the formation the murine telencephalon, while single mutants displayed normal telencephalic development [52]. We found all four FGF receptors expressed in spinal motor neurons innervating limb and axial musculature. While we did not detect an up-regulation of one of the three remaining receptor genes after elimination of *FGFR2*, compensation still might occur via one or more of these receptor molecules in combination with promiscuous signaling by FGFs in the limb mesenchyme. Isoforms of FGF8, for example, can bind to all four FGF receptor molecules [53], [54], and FGF10 is, next to limb bud induction, critically involved in lung formation by interacting with FGFR3 and FGFR4 [55]. Combinatorial elimination of several FGFRs in spinal motor neurons therefore is indispensable to further investigate the role of FGF receptor signaling for axon elongation and dorsal-ventral pathfinding.

Elimination of FGFR2b signaling in the entire organism was shown to lead to defective dental axon patterning caused by down-regulation of the repulsive axon guidance cue *Sema3A* in the tooth epithelium [34]. In chicken embryos, intrinsic expression of *Sema3A* critically influences fasciculation of motor axons and local availability of the axon guidance receptor Neuropilin-1 [35]. We found no obvious de-regulation of *Sema3A* expression by somatic motor neurons, which corresponds with our findings that the fasciculation of nerves that innervate the embryonal forelimb is not perturbed upon elimination of *FGFR2* in somatic motor neurons and argues against a direct regulation of *Sema3A* expression by FGFR2 signaling. Differential expression of *FGFR2* in subsets of motor neurons in the ventral horn of the spinal cord, however, still might be involved in the formation of target-specific motor pools at later stages of embryonal development. It has been shown that neurotrophins in the target musculature regulate the expression of transcription factors implicated in motor axon targeting decisions and sensory-motor connectivity: *PEA3* and *Er81*, both members of the ETS transcription factor family, are expressed in proprioceptive sensory neurons and specific motor pools that innervate the same targets [56], [57]. Loss of Er81 and PEA3 function does not influence generation of axonal projections and early axon pathfinding decisions, but branching of nerves in distal target regions, indicating that target derived signals influence later targeting and sensory-motor connectivity [56]. FGF2, a ligand of FGFR2 [20], is expressed in the limb mesenchyme and was shown to promote the phosphorylation and thus activation of the ETS domain containing transcription factor Elk3 by the Ras-Erk signaling pathway [58]. Also PEA3 has been identified as a downstream target of FGFR signaling [59], [60], thus, analysis of the activation of ETS transcription factors might reveal whether FGF signaling from the limb mesenchyme is involved in activation of genes that promote neuronal survival and formation of motor pools. FGF signaling was also shown to up-regulate *GDNF* and *NGF* mRNA in hippocampal neurons expressing *FGFR1* and *FGFR2* [61], [62], thereby contributing to the maintenance of connections within the central nervous system. While specific ablation of *FGFR2* only in motor neurons has no effect on early axon outgrowth, extension, guidance fidelity and fasciculation, it still might be involved in the establishment of specific distal nerve branches and maintenance of peripheral connections to limb musculature at later stages of embryonal and early postnatal development.

Olig2 is a basic helix-loop-helix transcription repressor that is expressed in the pMN domain where motor neurons and later also oligodendrocytes are generated. At the embryonic time points we have analyzed here, *Olig2* expression is specific for motor neurons in the spinal cord, and thus only ablates the FGF receptor in somatic motor neurons that send their axons to muscles in the periphery. At later time points, expression of *Olig2* is of critical importance for the development of oligodendrocytes in the brain and spinal cord (reviewed in [63]). As mentioned above, lack of FGFR2 signaling leads to a reduced number of oligodendrocytes in the ventral forebrain [42]. To what extent ablation of *FGFR2* by *Olig2-Cre* interferes with the generation and migration of oligodendrocytes or the subsequent insulation and trophic support to axons at spinal levels, still needs to be investigated.

Taken together, FGFR2 plays no direct, cell-autonomous role in motor neurons during early axon extension, fasciculation and targeted growth to specific limb muscles, however, later functions, e.g. in maintenance of axonal projections and support by sheathing glia cells cannot be ruled out.

## Materials and Methods

### Ethics Statement

Animals were handled and housed according to the federal guidelines for the use and care of laboratory animals, approved by the Helmholtz Zentrum München Institutional Animal Care and Use Committee and the Regierung von Oberbayern.

### Mouse Embryo Preparation

The genotype of mouse embryos was determined as described for *Hb9::eGFP* [43] and *Olig2-Cre* [41].The conditional allele of *FGFR2* (*FGFR2^flox/flox^* [40]) was identified with the forward primer (CCT CCT ACT ACA ATT CCA CC) and reverse primer (CCA GAG GGA ATA TGT GTT TT) with the following cycling parameters: 5 min preheating at 94°C, 35 cycles of denaturation at 94°C for 30 seconds, annealing of the primers at 51°C for 40 seconds and 1 min polymerization at 72°C. In all experiments, mutant mice (*FGFR2^flox/flox^; Cre^+^*) were compared to control littermates (*FGFR2^+/+^;Cre^+^ or FGFR^+/flox or flox/flox^;Cre^−^*). Day of vaginal plug was considered E0.5. n = 3 for all analyzed genotypes, if not stated differently.

### Immunohistochemistry

The protocols for wholemount embryo staining and immunohistochemistry have been described previously [44], [64], [65]. The following primary antibodies were used for fluorescent immunohistochemistry on cryosections of E10.5 to E12.5 embryos or for wholemount embryo preparations of E10.5 to E12.5 embryos: mouse anti-neurofilament 2H3 and mouse anti-Isl1/2 39.4D5 (obtained from the Developmental Studies Hybridoma Bank (DSHB) developed under the auspices of the NICHD and maintained by The University of Iowa, Department of Biological Sciences, Iowa City, IA 52242), goat anti-FoxP1 (R&D Systems) and rabbit anti-GFP (Invitrogen). Antibody staining was visualized using fluorochrome-conjugated secondary antibodies (1∶250; Molecular Probes; Jackson Dianova). For wholemount imaging, embryos were cleared using BABB (1 part benzyl alcohol, 2 parts benzyl benzoate) and imaged using a LSM510 Zeiss confocal microscope. Confocal stacks through the entire extent of the region of interest were acquired and collapsed on a single plane for further investigation.

### *In situ* Hybridization

*In situ* hybridization was performed as described previously [44], [64]–[66]. The *FGFR2* containing plasmid was a kind gift from Clive Dickson, and antisense probes were created using BamHI and T7 polymerase. The *FGFR1*, *FGFR3*, and *FGFR4* containing plasmids were kind gifts from Nilima Prakash. To quantify the differential expression of *FGFR2* in motor neurons of the LMCm and LMCl, FoxP1^+^/Isl1^+^ and FoxP1^+^/Isl1^−^ motor neurons, respectively, were counted on 12 µm coronal sections of E12.5 mouse embryos. The cells in the sub columns of the LMC defined by FoxP1 and Isl1 immunohistochemistry were analyzed for an *in situ* hybridization signal and cells showing *FGFR2* expression were counted to calculate the percentage of LMCm and LMCl cells expressing the FGF receptor. Significance was calculated using the two-tailed Student’s t-test.

### Quantification of Expression Levels

To quantify the expression levels of *FGFR1–4,* the area of the LMC (FoxP1^+^) and MMC (FoxP1^−/^Isl1^+^) was selected based on immunohistochemistry against FoxP1 and Isl1. Micrographs of *in situ* hybridization against *FGFR1–4* on 12 µm sections of E12.5 control and mutant embryos were transformed to grayscale and colors were inverted for quantification. The mean gray value in the determined area of the LMC or MMC was measured using Image J Software and normalized to the mean gray value of the white matter of the spinal cord on the same section. Significance was calculated using the two-tailed Student’s t-test.

### Quantification of Pre-plexus Fasciculation

To quantify defasciculation of motor and sensory fibers before the plexus region in E10.5 wholemount embryos, the individual thickness of the 6 spinal nerves contributing to the forelimb-plexus was measured (“a” in Fig. 3C), summarized, and normalized to the length of the spinal cord from which these 6 projections originate (“b” in Fig. 3C) to determine a fasciculation coefficient [44]. “a” was furthermore used to quantify the individual thickness of spinal nerves contributing to the brachial plexus. Both sides of the embryos were analyzed. Significance was calculated using the two-tailed Student’s t-test.

### Quantification of Distal Advancement

To quantify the distance of ingrowth of motor axons into the forelimb of E12.5 embryos, the length of the distal-most motor fiber was measured starting from the reference point and normalized with the length of the forelimb (see Fig. 3I for a schematic showing of the reference point and the lengths measured). Both forelimbs were analyzed. Significance was calculated using the two tailed Student’s t-test.

### Retrograde Labeling of Neurons

Dextran-conjugated Rhodamine was injected into dorsal limb musculature of E12.5 embryos, which is normally innervated by motor neurons of the LMCl. Preparations were incubated for 4 hours in DMEM/F12 aerated with 5% CO_2_ in 95% O_2_ (Carbogen) prior to 1 hour of fixation in 4% PFA in PBS and cryoprotection in 30% sucrose in PBS and then cryosectioned at 12 µm. To quantitate misprojecting neurons, backfilled Rhodamin^+^ neurons were counted, and the percentage of aberrantly projecting neurons was calculated based on immunostaining against Isl1 (LMCm). Significance was calculated using the two-tailed Student’s t-test.
